# Assessment of Major Neurocognitive Disorders in Primary Health Care: Predictors of Individual Risk Factors

**DOI:** 10.3389/fpsyg.2020.01413

**Published:** 2020-06-17

**Authors:** Susana Sousa, Laetitia Teixeira, Constança Paúl

**Affiliations:** ^1^Abel Salazar Institute of Biomedical Sciences – University of Porto (ICBAS.UP), Porto, Portugal; ^2^Center for Health Technology and Services Research (CINTESIS.ICBAS), Porto, Portugal; ^3^Office on Ageing Issues 50^+^ (CA50^+^), Porto, Portugal

**Keywords:** major neurocognitive disorders, predictors, primary health care, older people, health

## Abstract

Major Neurocognitive Disorders describe the symptoms of a large group of diseases causing a progressive decline in individual’s functioning. It is an umbrella term describing a decline in memory, intellectual ability, reasoning, and social skills, as well as changes in normal emotional reactions. The general practitioner is instrumental in the early diagnosis of Major Neurocognitive Disorder. Individual risk factors act as contributing variables affecting the probability of someone developing a Major Neurocognitive Disorder and may be considered predictive factors. This study aimed (i) to show the utility of using the Global Deterioration Scale in primary health care settings as a measure to assess the stage of cognitive function for individuals identified with Major Neurocognitive Disorders and (ii) to identify predictors of severe Major Neurocognitive Disorders. Potential predictors of Major Neurocognitive Disorders considered in this study were: sex, age, years of education, social isolation, hearing impairment, cardiovascular disease, hypertension, diabetes, smoking habits, alcohol consumption, physical activity, hand strength, and nutritional status. The sample comprised 250 adults, 30.4% were classified as having probable Major Neurocognitive Disorder. The variables significantly associated with probable Major Neurocognitive Disorder were age, years of education, hearing impairment, cardiovascular disease, hand strength, nutritional status, and physical activity. In the multivariable model, only age, education, physical activity and hand strength remained significant predictors of probable Major Neurocognitive Disorder. The Global Deterioration Scale seems to be a usefull instrument in primary healthcare settings, as it guides the general practitioner in observing the patients’ cognitive functioning. Advanced age, lower education, lower hand strength and absence of physical activities should be taken into account as they increase the chance of severe Major Neurocognitive Disorders. Primary health care providers, including general practitioners are very important in the diagnosis and follow up of Major Neurocognitive Disorder. The general practitioner is in most cases the patients’ first and for many patients the only contact, thus having a critical role in evaluating with caution what is part of normal or pathological aging, and the individual factors that can increase the likelihood of developing Major Neurocognitive Disorder to further support patients in the course of the disease.

## Introduction

Major Neurocognitive Disorder (MND) – previously called dementia – is a syndrome that progresses with significant deterioration of cognitive domains as compared to previous levels of cognitive performance in memory, speech, reasoning, intellectual function, and/or spatiotemporal perception, and may also be associated with changes in emotional behavior and difficulties at the functional level. The decline is initially noticed by the individual, the family, or the General Practitioner (GP) who is usually responsible for the early diagnosis (American Psychiatric Association [APA], 2014).

MND may result from brain disorders, classified as primary (degenerative), or consequence of other conditions (secondary) (Emre, 2009). The most common types of MND are: Alzheimer’s disease, Vascular dementia, Lewy body dementia and Frontotemporal dementia. In secondary MND (e.g., alcoholic dementia, infectious diseases) the symptoms may be treated and/or prevented. Therefore, a correct diagnosis is crucial. This is supported by a detailed collection of the person’s clinical history, neurological and neuropsychological examination and the comprehensive use of laboratory and imaging tests. In primary MND, early diagnosis is equally crucial either to delay the progression of cognitive symptoms and to control/stabilize psychiatric manifestations (Ribeira et al., 2004).

Some symptoms of MND might be confused with typical changes occurring in healthy aging. The first signs of MND are very subtle and vague, and can be difficult to detect. Those signs are also very diverse and, as such, we must do a staging of Dementia, which is not only centered on aspects of the cognitive forum (Fernández-Ballesteros et al., 2012).

The GP is instrumental in the detection of the first signals of MND. Additionally, the GP supports the persons with MND and their caregivers in organizing and planning interventions at an early stage of the disease and care provision as the disease progresses (Sequeira, 2010). To confirm any suspicion on the decline in cognitive functioning of a patient, the GP needs to use a screening instrument that should be easy and quick to apply. The most common practice is the use of the Mini Mental State Examination (MMSE) (Folstein et al., 1975) that has been used to detect and monitor the evolution of cognitive impairment (Valle et al., 2009). The disadvantage of using MMSE, however, is the fact that it does not allow to establish stages of cognitive function or detect early stages of cognitive decline.

The Global Deterioration Scale (GDS), developed by Barry Reisberg (1988), provides an overview of the stages of cognitive function for those living with a primary degenerative dementia. This instrument is easy to use and facilitate the assessment of subjective cognitive complaints (Custodio et al., 2017). GDS stages are associated with cognitive function but also with basic and instrumental activities of daily living (ADL; e.g., dressing, eating, and bathing) and instrumental activities of daily living (IADL; e.g., handling finances, medication management (Paul et al., 2002). GDS is not a diagnostic scale and was developed as a qualitative severity rating only (Hartmaier et al., 1994; Brooke and Bullock, 1999; Petersen et al., 1999). According to Custodio et al. some studies validate the GDS as an assessment tool to detect mild cognitive impairment.

The GDS includes seven stages: Stage 1 (no cognitive decline) – No subjective or objective memory deficits. Stage 2 (Very Mild Cognitive Decline) – Subjective complaints of memory deficit, but no objective measurements of memory deficit. Stage 3 (Mild Cognitive Decline) – The individual now meets criteria for mild cognitive impairment. Stage 4 (Moderate Cognitive Decline) – The individual is now classified as being mildly demented. This could manifest as a clear deficit on concentration, handling finances, orientation, and recognition of time and place. Symptoms such as flattening of affect and anxiety start to occur. Stage 5 (Moderately Severe Cognitive Decline) – The individual now meets criteria for moderate dementia and can no longer function without some assistance but can toilet and eat on their own. Stage 6 (Severe Cognitive Decline) – The individual meets criteria for moderately severe dementia. The individual is entirely dependent on someone else for survival and are generally unaware of their surroundings, year, season, etc. Personality and emotional changes occur. Stage 7 (Very Severe Cognitive Decline) – The individual is now severely demented. The individual has lost all verbal abilities and is incontinent, as well as basic psychomotor skills (Hardcastle et al., 2019).

### Predictive Factors of MND

MND is likely to develop in a continuous process (Brooks and Loewenstein, 2010). Individual factors affect the likelihood of developing MND. Those factors predicting the development of the disease should be known, and preventive interventions must build on this knowledge.

Previous studies have identified predictive factors of MND, which can be grouped into sociodemographic (e.g., sex, age, and years spent in education and social isolation), health factors (e.g., hearing loss, cardiovascular diseases, hypertension, diabetes, handgrip strength, and nutritional status) and bio-behavioral factors (e.g., smoke, alcohol, and physical activity) (Helzner et al., 2009; Nagai et al., 2010; Polidori et al., 2012; Baumgart et al., 2015; Santana et al., 2015; Schwarzinger et al., 2018). Given that most of these factors are potentially modifiable (e.g., diabetes, cholesterol, depression, or malnutrition; Chen et al., 2017), the individual can play an active role in the development of the disease, allowing for more efficient intervention. Primary prevention in the primary health care context is very important for the course of MND, and should focus on the identification of situations that increase the likelihood of occurrence or worsening of symptoms. However, few studies identify predictive factors associated with severe stage of MND (Eshkoor et al., 2016).

The objectives of this paper are: (i) to show the utility of using the GDS in primary health care settings as a measure to assess the stage of cognitive function for individuals identified with MND (ii) to identify predictors of severe MND.

## Materials and Methods

### Participants

This study is an observational cross-sectional study that is part of a larger project aiming at “Needs of Care for People with Dementia.”

The inclusion criteria defined in the largest project, also used in this study, are: (i) to be a user of a primary care unit in the area of Portuguese North Regional Health Authority (ARS Norte); (ii) age 65 years or plus; (iii) living in the community; (iv) presence of mental health concerns. The exclusion criteria were as follows: (i) patient not using a primary health-care unit covered by the ARS North; (ii) age less than 65 years old; (iii) living in nursing home, hospital or psychiatric institution; and (iv) absence of memory concerns (patients classified in stage 1 of the GDS).

### Instruments

The study protocol was based on the “Community Assessment of Risk and Treatment Strategies (CARTS) Program” developed in the University College Cork, Ireland (Caoimh et al., 2012). The protocol is divided in three different sections: The purpose of the first part (Part A) was to assess the patient with probable dementia referred by the health professional (GP or nurse); the second part (Part B) was used to assess the patient with probable dementia by the GP; the final part (Part C) focus the evaluation of the informal caregiver of the patient with probable dementia (if available).

In this study, information provided in Part A and B of the assessment protocol was used. Data were collected by resorting to the following instruments:

*Sociodemographic questionnaire:* It allows to collect data about the patient with probable dementia, including sex (M/F), age, years spent in education, and social isolation (living with someone/living alone).

*Cognition:* Global Deterioration Scale (GDS) (Reisberg et al., 1982, portuguese version; Leitão et al., 2007). This instrument allows to qualitatively classify the individuals according to the stage of primary degenerative dementia. This scale has been validated with behavioral, neuroanatomic, and neurophysiological measures in patients with primary degenerative dementia. GDS includes seven different stages of patient classification (see section “Introduction”). An overall description of the symptoms and clinical characteristics expected for each stage of dementia is provided in the instrument, and such descriptions are considered for deciding on the most appropriate global level (stage) of cognition and function.

*Health:* Older Americans Resources and Services (OARS) (Fillenbaum and Smyer, 1981, portuguese version; Rodrigues, 2008) is a program of resources and services for old people. The OARS methodology was developed to assess functional capacity in five key areas for older adults’ quality of life: social resources, economic resources, mental health, physical health, and activities of daily living. It also measures the use and perceived need for various types of services, enabling the evaluation of intervention programs and informed decision-making on the impact of resources and services. This instrument contains a list of the most common problems in older people and this study considered cardiovascular problems, hypertension, diabetes, hearing loss, and dementia; Handgrip strength was assessed using a dynamometer considering four attempts, two on each hand. The final score corresponds to the average of the highest values for each hand (Wearing et al., 2018; Zammit et al., 2019).

*Bio-behavioral aspects:* Frequency of physical activity [(1) more than once a week; (2) once a week; (3) 1–3 times per month; (4) almost never or never]; Alcohol and tobacco consumption [(1) no; (2) yes, but stopped; (3) yes]; Short-Term Nutritional Assessment (MNA-SF) (Rubenstein et al., 2001) is a nutritional screening and assessment tool aimed at identifying malnourished patients. It consists in six questions and the total score ranges from 0 to 14. A score of 11 or above indicates possible malnutrition.

### Procedures

The Risk Instrument for Screening in the Community (Caoimh et al., 2012) was first used as a screening tool to identify potential participants, i.e., patients with mental health concerns. Based on these results, and considering strata by age group, sex, and region, 572 participants with mental health concerns were randomly selected. Of these, 504 agreed to participate and 436 were eligible to participate. The final sample of this study included 250 individuals with mental health concerns and with the evaluation provided by the GP (Part B of the study protocol).

The data collection lasted 27 months (from January 2014 to April 2016). The Part A of the study protocol was administered to potential participants by trained interviewers and took on average 45 min to complete. Most interviews were carried in health-care centers, and, when participants were not able to meet the interviewers at the health centers, interviews were completed at patients’ home.

After the first interview, the GP completed the Part B of the evaluation protocol using mainly the existing clinical registries of the patient. To complete the checklist of diagnoses (OARS), the GP used the International Classification of Diseases 9th Revision Clinical Modification (ICD-9-CM). This classification was adopted in Portugal in 1989 for the purposes of clinical coding. In the specific case of the diagnosis of dementia, the coding F03-dementias (not specified) was considered.

The study was submitted to the Ethics Committee of the ARS-Norte and was approved unanimously on January 7, 2014 (Reference No. 6/2014). All participants signed the informed consent form complying with the Declaration of Helsinki.

The detailed methodological aspects are reported and can be consulted elsewhere (Teixeira et al., 2017).

### Statistical Analysis

First, a receiver operating characteristic (ROC) analysis was performed to determine the optimal GDS cutoff point to identify stages of MND, considering the GP diagnosis as gold standard [coding F03-dementias (not specified) in the diagnosis’ checklist]. The area under the curve (AUC) was calculated as well as the sensitivity and specificity values. The Youden Index (Sensitivity + Specificity-1) was used to obtain the optimal cutoff point.

Then, based on the optimal cutoff point obtained, two groups were considered: patients with non-severe MND vs. patients with severe MND.

Descriptive analysis of the data was performed in order to describe the sociodemographic and health profile of the study sample. Differences between groups with and without non-severe MND across sociodemographic, health, and bio-behavioral variables were assessed using the Student *t*-test (for continuous variables) and the Chi-Square test (for categorical variables). To identify potential predictors of severe MND, a multivariable binary logistic regression model was used.

All analyses were performed using IBM SPSS Statistics 24. A significance level of 0.05 was considered.

## Results

**FIGURE 1:**
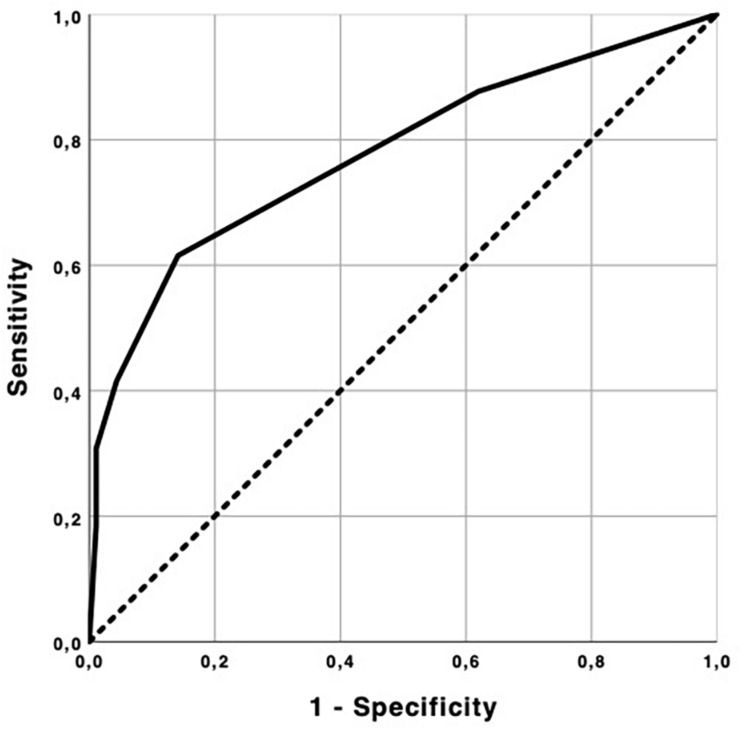
ROC curve.

In order to identify stages of MND through the optimal GDS cutoff point, we use a ROC curve analysis. The area under the ROC curve [AUC = 0.777, 95% CI = (0.700; 0.854)] shows that GDS can predict about 77.7% of the events (severe MND). Given the estimates for sensitivity and specificity (0.615 and 0.860, respectively) and based on the Youden index, the optimal cutoff was 3.5, i.e., individuals with a score equal or greater than four were classified as severe MND (Figure 1).

With this cutoff point we classified and grouped the individuals in the sample as “with non-severe MND” or “with severe MND.”

More than half of the sample (*N* = 250) is female, and the average age 76 years old. The participants spent, on average, 2.5 years in formal education and only a small percentage live alone. About 1/3 of the participants have hearing impairment and more than 40% have diabetes, cardiovascular problems or hypertension. The average handgrip strength and nutritional status score is below 20%. Regarding the bio-behavioral aspects, more than 50% of the sample do not smoke, report to exercise more than once a week and less than 50% do not drink alcohol (Table 1).

**TABLE 1:** Characteristics of the total sample and according groups.

		**Total**	**Non-severe MND**	**Severe MND**	***p***	**OR**
		***n* (%) or mean (*SD*)**	***n* (%) or mean (*SD*)**	***n* (%) or mean (*SD*)**		
Sex	Male	111 (44.4)	80 (72.1)	31 (27.9)	0.448	Ref
	Female	139 (55.6)	94 (67.6)	45 (32.4)		1.235
Age		76.14 (7.3)	74.39 (6.5)	80.17 (7.2)	<0.001	1.125
Years education		2.85 (2.0)	3.31 (1.9)	1.79 (1.8)	0.006	0.631
Social isolation	Living with someone	212 (85.5)	147 (69.3)	75 (30.2)	0.728	1.150
	Living alone	36 (14.5)	26 (72.2)	10 (27.8)		Ref
Hearing loss	With problems	53 (23.2)	28 (52.8)	25 (47.2)	0.002	2.658
	Without problems	175 (76.8)	131 (74.9)	44 (25.1)		Ref
Cardiovascular diseases	Yes	102 (45.9)	58 (56.9)	44 (43.1)	<0.001	2.883
	No	120 (54.1)	95 (79.2)	25 (20.8)		Ref
Hypertension	Yes	188 (78)	128 (68.1)	60 (31.9)	0.616	1.187
	No	53 (22)	38 (71.7)	15 (28.3)		Ref
Diabetes	Yes	98 (42.1)	69 (70.4)	29 (29.6)	0.712	0.899
	No	135 (57.9)	92 (68.1)	43 (31.9)		Ref
Hand strength		19.6 (8.9)	21.2 (9.1)	15.5 (7.1)	<0.001	0.914
Nutritional status		10.7 (2.6)	11.41 (2.2)	9.11 (2.9)	<0.001	0.713
Smoke	No	131 (79.4)	89 (67.9)	42 (32.1)	0.255	Ref
	Yes, but I stopped	29 (17.6)	24 (82.8)	5 (17.2)		0.441
	Yes	5 (3)	4 (80)	1 (20)		0.530
Alcohol consumption	No	68 (41.7)	41 (60.3)	27 (39.7)	0.108	Ref
	Only on very special occasions	14 (8.6)	11 (78.6)	3 (21.4)		0.414
	Occasionally	26 (16)	21 (80.8)	5 (19.2)		0.362
	Yes	55 (33.7)	42 (76.4)	13 (23.6)		0.470
Physical activity	More than 1x/week	146 (59.1)	123 (84.2)	23 (15.8)	<0.001	Ref
	1x/week	17 (6.9)	13 (76.5)	4 (23.5)		1.645
	1–3x/month	8 (3.2)	7 (87.5)	1 (12.5)		0.764
	Almost never or never	76 (30.8)	30 (39.5)	46 (60.5)		8.200

The potential predictors of severe MND considered in this study were: gender, age, years of education, social isolation, hearing loss, cardiovascular disorders, hypertension, diabetes, smoking, alcohol consumption, physical activity, hand strength, and nutritional status.

Of the referred factors, there was a significant association with severe MND for age (*p* < 0.001), years of education (*p* = 0.006), hearing loss (*p* = 0.002), cardiovascular disorders (*p* < 0.001), hand strength (*p* < 0.001), nutritional status (*p* < 0.001), and physical activity (*p* < 0.001).

Individuals with severe MND had a higher mean age and lower years of education compared to individuals with non-severe MND. Additionally, the percentage of individuals with severe MND was higher in individuals with hearing and cardiovascular problems. Individuals with severe MND had a lower mean of Hand Strength and a lower mean of MNA score. Finally, individuals who exercise more than once a week have a lower percentage of severe MND than individuals who never exercise.

In order to identify independent predictors of severe MND, we used a multivariable binary logistic regression model, considering results obtained from the bivariate analysis (Table 1). Only age, years of education, physical activity and hand strength have shown to be significant predictors of severe MND (see Table 2).

**TABLE 2:** Multivariable logistic regression model.

**Predictors**		**OR**	**95% CI**	***p***
Sex	Male	1	–	–
	Female	1.282	0.430–3.829	0.656
Age		1.090	1.018–1.168	0.014
Years of education		0.696	0.550–0.882	0.003
Physical activity	More than once a week	1	–	–
	About once a month	0.917	0.234–3.590	0.901
	Almost never or never	4.121	1.635–10.39	0.003
Hand strength		0.919	0.856–0.987	0.020
Nutritional status		0.954	0.794–1.146	0.613
Cardiovascular diseases	No	1	–	–
	Yes	2.164	0.892–5.246	0.088
Hearing loss	No	1	–	–
	Yes	0.745	0.268–2.072	0.573

Older patients had more chances to had severe MND (*OR* = 1.090; 95% CI 1.017–1.167). Additionally, the more years of education the participants had, the lower the chance of having been classified with severe MND (*OR* = 0.696; 95% CI 0.550–0.882). Similar results were found for hand strength, with higher hand strength related with a decreased risk of severe MND (*OR* = 0.919; 95% CI 0.856–0.986). Finally, regarding physical activity, those who almost never or never practice physical exercise had a higher chance of being classified as having severe MND than those who never practice physical exercise (*OR* = 4.121; 95% CI 1.635–10.390).

## Discussion

The first objective of this study related to the need of identification of MND stages of MND by GPs, to facilitate an early referral of patients to specific and beneficial interventions. This would enable to timely implement appropriate interventions targeted at these patients and their caregivers and aimed at monitored more effectively the disease from its outset and during its course. There is no specific protocol to make the diagnosis of MND in Primary Health Care settings. GPs tend to use various tests and complementary exams, whenever available, to determine whether symptoms meet diagnosis criteria of MND and to exclude other possible causes for observed symptoms.

Although there are other scales widely used, such as the “Clinical Dementia Rating scale (CDR)” and the “Clinical Dementia Score” (Morris, 1991) we have selected the GDS accounting for the fact that this is a friendly tool that allows the GP to go further with the diagnosis and classify the state of the MND, through observational interviewing and recording of the patients’ symptoms. In addition to the usefulness of this instrument to appraise the stage of MND, thus focusing mainly on cognition, it is one of the simplest scales, helping to understand the patients’ actual and future condition, and proved to be very suitable. We determined the optimal cutoff point for the GDS in the early diagnosis of probable MND, considering the medical diagnosis as gold standard. We have determined that individuals with a GDS score equal or greater than four are considered as having severe MND.

Having as a health priority the early diagnosis of MND and the classification of the stage of the disease in primary health care settings, the second aim of this study was to investigate the predictors of MND, with the ultimate goal of preventing/intervening in some risks that may be circumventable. It was possible to identify four predictors of MND: age, years spent in education, physical activity and hand strength. Physical activity, hand strength and education play a protective role (“the more the better”). On the other hand and as expected, while age increases, the risk of MND also increases.

The findings from this study on the risk factors for MND are in line with available literature on the topic. Regarding physical activity, other studies have suggested Weuve et al. (2004) that regular physical activity reduces vascular risk factors and may directly increase the production of neurotrophic factors in the brain physical exercise as a protective function of neurons. Regarding the role of education, some studies (Amieva et al., 2014) report that the mechanism through which more educated individuals are at lower risk of developing MND is the greater ability of more educated individuals to cope with symptoms.

The older the person, the greater the risk of having MND. Age is the main risk factor for MND. After the age of 65, the risk of MND increases every 5 years. The same is true for hand strength: the lower the strength, the higher the risk of MND. Among older adults, this association is often cited for its relation to the concept of frailty and implications on the person’s functional status (Abizanda et al., 2012). Several studies (Jang and Kim, 2015) have found a significant association of cognitive decline with worse hand strength among older adults values in the elderly. Hand strength may represent an age-related change in physical function and frailty, contributing to cognitive decline and increasing the risk of MND. Thus, we can formulate the hypothesis that cognitive changes may influence the motor skills of older adults, which would justify the worse performance in the hand strength test in older persons with cognitive deficit. Another justification would be that that low hand strength is a consequence of inactivity, which can contribute to cognitive decline. In any case, hand strength losses arean alert sign to the development of MND.

Although significant contribution of sex was not found in this study, the literature has been suggesting that female are at greater risk of developing MND than male. Worldwide, most people with MND or at risk of developing MND are women, according to Alzheimer’s Disease International (2015). However, other studies suggest that, up to the age of 90, there is no sex differences in the incidence of MND, above this age men appear to be at lower risk than women (Ruitenberg et al., 2001).

In future studies, other variables should be taken into account and investigated about their association with the development of MND. Sleep hygiene, for instance, is an important variable. Some studies suggest that sleep changes often occur in people with MND, and can aggravate with the progression of MND. In addition to normal sleep changes as a result of aging, changes that occur in the brain increase sleep disorders in older adults with MND (Rose and Lorenz, 2012). Changes in the pattern of sleep modify the homeostatic balance, with repercussions on psychological function, immune system, performance, behavioral response, mood and ability to adapt (Ebersole and Hess, 2001).

The main limitations of this study are related to its cross-sectional design, not allowing the observation of the disease progression as classified by GDS. Moreover, the GDS may not be very sensitive to cognitive changes over time. Also, while the coding system for the diagnosis of dementia is unique both at national and international levels, the GPs follow different protocols to assess patients and stablish the diagnosis that was used as a golden standard in this study. Other concerns are the dimension of the sample and the heterogeneity of this population (in terms of age, education, access to health services and even life style) making it difficult to generalize the results. However, this study is innovative because it is based in a Portuguese representative sample of users of the health care centers in the north of the country, and reports on current MND diagnosis by GPs. These findings have clinical relevance and implications for case management in dementia in the context of primary health care.

## Conclusion

Primary health care settings are very important in the identification of MND. The GP is in most cases the patients’ first and only contact and for this professional the differentiation between normal or pathological aging should be clear and the individual factors that can contribute to MND must be known. The recognition of the stage of MND supports a more accurate understanding of the patient, family conditions and needs during the progression of the disease and should lead to an adequate customization of available health and social support services. An early diagnosis of MND, together with the use of GDS to acknowledge the stage of the disease in which the patient is, and the identification of predictors of probable MND will consubstantiate very relevant aspects of clinical practice. These aspects are the foundation of the design of more targeted interventions for each individual, which at should emphasize physical and lifelong learning throughout life.

## Data Availability Statement

The datasets for this article are not publicly available because: this study is part of a larger study. Requests to access the datasets should be directed to CP, paul@icbas.up.pt.

## Ethics Statement

The studies involving human participants were reviewed and approved by the Ethics Committee of the ARS-Norte on January 7, 2014 (Reference No. 6/2014). The patients/participants provided their written informed consent to participate in this study.

## Author Contributions

SS wrote the manuscript and conducted the data analysis. LT and CP reviewed the manuscript. All authors contributed to the manuscript and approved the submitted version.

## Conflict of Interest

The authors declare that the research was conducted in the absence of any commercial or financial relationships that could be construed as a potential conflict of interest.

## References

[B1] Abizanda P. Navarro J. L. García-Tomás M. I. López-Jiménez E. Martínez-Sánchez E. Paterna G. (2012). Validity and usefulness of hand-held dynamometry for measuring muscle strength in community-dwelling older persons. *Arch. Gerontol. Geriatr.* 54 21–27. 10.1016/j.archger.2011.02.006 21371760

[B2] Alzheimer’s Disease International (2015). *Women and Dementia: A Global Research Review.* London: Alzheimer’s Disease International.

[B3] American Psychiatric Association [APA] (2014). *Manual de Diagnóstico e Estatística Das Perturbações Mentais*, 5. a Edn Lisboa: Climepsi Editores.

[B4] Amieva H. Mokri H. Le Goff M. Meillon C. Jacqmin-Gadda H. Foubert-Samier A. (2014). Compensatory mechanisms in higher-educated subjects with Alzheimer’s disease: a study of 20 years of cognitive decline. *Brain* 137 1167–1175. 10.1093/brain/awu035 24578544

[B5] Baumgart M. Snyder H. M. Carrillo M. C. Fazio S. Kim H. Johns H. (2015). Summary of the evidence on modifiable risk factors for cognitive decline and dementia: a population-based perspective. *Alzheimer’s Dement.* 11 718–726. 10.1016/j.jalz.2015.05.016 26045020

[B6] Brooks L. G. Loewenstein D. A. (2010). Assessing the progression of mild cognitive impairment to Alzheimer’s disease: current trends and future directions. *Alzheimer’s Res. Ther.* 2:28. 10.1186/alzrt52 PMC2983437 20920147

[B7] Brooke P. Bullock R. (1999). Validation of a 6 item cognitive impairment test with a view to primary care usage. *Int. J. Geriatr. Psychiatry* 14 936–940. 10.1002/(SICI)1099-1166(199911)14:11<936::AID-GPS39>3.0.CO;2-1 10556864

[B8] Caoimh R. O. Healy E. Connell E. O. Gao Y. Molloy D. W. (2012). The Community Assessment of Risk Tool,(CART): investigation of inter-rater reliability for a new instrument measuring risk of adverse outcomes in community dwelling older adults. *Irish J. Med. Sci.* 181 S227–S227.

[B9] Chen L. Y. Wu Y. H. Huang C. Y. Liu L. K. Hwang A. C. Peng L. N. (2017). Predictive factors for dementia and cognitive impairment among residents living in the veterans’ retirement communities in Taiwan: Implications for cognitive health promotion activities. *Geriatr. Gerontol. Int.* 17 7–13. 10.1111/ggi.13039 28436185

[B10] Custodio N. Becerra-Becerra Y. Alva-Diaz C. Montesinos R. Lira D. Herrera-Pérez E. (2017). Validación y precisión de la escala de deterioro global (GDS) para establecer severidad de demencia en una población de Lima. *Ces Med.* 31 14–26. 10.21615/cesmedicina.31.1.2

[B11] Ebersole P. Hess P. A. (2001). *Geriatric Nursing & Healthy Aging.* Maryland Heights, MI: Mosby Incorporated.

[B12] Emre M. (2009). Classification and diagnosis of dementia: a mechanism-based approach. *Eur. J. Neurol.* 16 168–173. 10.1111/j.1468-1331.2008.02379.x 19146638

[B13] Eshkoor S. A. Hamid T. A. Shahar S. Ng C. K. Mun C. Y. (2016). Predictive fac-tors of severe stage of dementia among the Malaysian elderly. *Arch. Gerontol. Geriatr. Res.* 1 6–12.

[B14] Fernández-Ballesteros R. Botella J. Zamarrón M. D. Molina M. Á Cabras E. Schettini R. (2012). Cognitive plasticity in normal and pathological aging. *Clin. Interv. Aging* 7:15. 10.2147/cia.s27008 22291469 PMC3267402

[B15] Fillenbaum G. G. Smyer M. A. (1981). The development, validity, and reliability of the OARS multidimensional functional assessment questionnaire. *J. Gerontol.* 36 428–434. 10.1093/geronj/36.4.428 7252074

[B16] Folstein M. F. Folstein S. E. McHugh P. R. (1975). “Mini-mental state”: a practical method for grading the cognitive state of patients for the clinician. *J. Psychiatr. Res.* 12 189–198. 1202204 10.1016/0022-3956(75)90026-6

[B17] Hardcastle C. Taylor B. Price C. (2019). *Global Deterioration Scale.* Toronto: Alzheimer Society of Canada.

[B18] Hartmaier S. L. Sloane P. D. Guess H. A. Koch G. G. (1994). The MDS Cognition Scale: A valid instrument for identifying and staging nursing home residents with dementia using the Minimum Data Set. *J. Am. Geriatr. Soc.* 42 1173–1179. 10.1111/j.1532-5415.1994.tb06984.x 7963204

[B19] Helzner E. P. Luchsinger J. A. Scarmeas N. Cosentino S. Brickman A. M. Glymour M. M. (2009). Contribution of vascular risk factors to the progression in Alzheimer disease. *Arch. Neurol.* 66 343–348. 19273753 10.1001/archneur.66.3.343 PMC3105324

[B20] Jang J. Y. Kim J. (2015). Association between handgrip strength and cognitive impairment in elderly Koreans: a population-based cross-sectional study. *J. Phys. Ther. Sci.* 27 3911–3915. 10.1589/jpts.27.3911 26834379 PMC4713818

[B21] Leitão O. Nina A. Monteiro I. (2007). “Escala de deterioração global,” in *Tradução e organização Escala e testes na demência*, Coords A. Mendonça M. Guerreiro (Portuguese: SciELO), 9–13.

[B22] Morris J. C. (1991). The Clinical Dementia Rating (CDR): Current version and scoring rules. *Young* 41 1588–1592. 10.1212/wnl.43.11.2412-a 8232972

[B23] Nagai M. Hoshide S. Kario K. (2010). Hypertension and dementia. *Am. J. Hypertens.* 23 116–124. 10.1038/ajh.2009.212 19927134

[B24] Paul R. H. Cohen R. A. Moser D. J. Zawacki T. Ott B. R. Gordon N. (2002). The global deterioration scale: relationships to neuropsychological performance and activities of daily living in individuals with vascular dementia. *J. Geriatr. Psychiatr. Neurol.* 15 50–54. 10.1177/089198870201500110 11936244

[B25] Petersen R. C. Smith G. E. Waring S. C. Ivnik R. J. Tangalos E. G. Kokmen E. (1999). Mild cognitive impairment: clinical characterization and outcome. *Arch. neurol.* 56 303–308. 10.1001/archneur.56.3.303 10190820

[B26] Polidori M. C. Pientka L. Mecocci P. (2012). A review of the major vascular risk factors related to Alzheimer’s disease. *J. Alzheimer’s Dis.* 32 521–530. 10.3233/jad-2012-120871 22836188

[B27] Reisberg B. Ferris S. H. de Leon M. J. Crook T. (1982). The Global Deterioration Scale for assessment of primary degenerative dementia. *Am. J. Psychiatry* 139 1136–1139. 7114305 10.1176/ajp.139.9.1136

[B28] Ribeira S. Ramos C. Sá L. (2004). Avaliação inicial da demência. *Rev. Portug. Med. Geral Fam.* 20 569–577.

[B29] Rodrigues R. M. C. (2008). Validação da versão em português europeu de questionário de avaliação funcional multidimensional de idosos. *Rev. Panamericana Salud Públ.* 23 109–115. 10.1590/s1020-49892008000200006 18371281

[B30] Rose K. M. Lorenz R. (2012). Sleep disturbances in dementia: What they are and what to do. *J. Gerontol. Nurs.* 36 9–14. 10.3928/00989134-20100330-05 PMC3062259 20438013

[B31] Rubenstein L. Z. Harker J. O. Salvà A. Guigoz Y. Vellas B. (2001). Screening for undernutrition in geriatric practice: developing the short-form mini-nutritional assessment (MNA-SF). *J. Gerontol. Ser.* 56 M366–M372. 10.1093/gerona/56.6.m366 11382797

[B32] Ruitenberg A. Ott A. van Swieten J. C. Hofman A. Breteler M. M. (2001). Incidence of dementia: does gender make a difference? *Neurobiol. Aging* 22 575–580. 10.1016/s0197-4580(01)00231-7 11445258

[B33] Santana I. Farinha F. Freitas S. Rodrigues V. Carvalho Á (2015). Epidemiologia da Demência e da Doença de Alzheimer em Portugal: Estimativas da Prevalência e dos Encargos Financeiros com a Medicação. *Acta Méd. Portug.* 28 182–188. 26061508

[B34] Schwarzinger M. Pollock B. G. Hasan O. S. Dufouil C. Rehm J. Baillot S. (2018). Contribution of alcohol use disorders to the burden of dementia in France 2008–13: a nationwide retrospective cohort study. *Lancet Public Health* 3 e124–e132. 10.1016/s2468-2667(18)30022-7 29475810

[B35] Sequeira C. (2010). *Cuidar de Idosos com Dependência Física e Mental.* Portugal: Lidel Edições Técnicas Lda.

[B36] Teixeira L. Dos Santos P. M. Alves S. Azevedo M. J. Duarte M. G. Leuschner A. (2017). Screening of Dementia in Portuguese Primary care: Methodology, assessment Tools, and Main results. *Front. Med.* 4:197. 10.3389/fmed.2017.00197 29181378 PMC5693885

[B37] Valle E. A. Castro-Costa É Firmo J. O. Uchoa E. Lima-Costa M. F. (2009). Estudo de base populacional dos fatores associados ao desempenho no Mini Exame do Estado Mental entre idosos: Projeto Bambuí. *Cadernos Saúde Públ.* 25 918–926. 10.1590/s0102-311x2009000400023 19347218

[B38] Wearing J. Konings P. Stokes M. de Bruin E. D. (2018). Handgrip strength in old and oldest old Swiss adults–a cross-sectional study. *BMC Geriatr.* 18:266. 10.1186/s12877-018-0959-0 PMC6219188 30400825

[B39] Weuve J. Kang J. H. Manson J. E. Breteler M. M. Ware J. H. Grodstein F. (2004). Physical activity, including walking, and cognitive function in older women. *JAMA* 292 1454–1461. 15383516 10.1001/jama.292.12.1454

[B40] Zammit A. R. Piccinin A. M. Duggan E. C. Koval A. Clouston S. Robitaille A. (2019). A coordinated multi-study analysis of the longitudinal association between handgrip strength and cognitive function in older adults. *J. Gerontol.* 11:gbz072. 10.1093/geronb/gbz072 PMC7813182 31187137

